# Glabridin Inhibits Melanogenesis and Melanin Transfer via Wnt/β-Catenin Pathway and Rho Family GTPase-Mediated Dendritic Formation Suppression

**DOI:** 10.3390/ph19030469

**Published:** 2026-03-12

**Authors:** Lili Li, Xiaoya Zhang, Guangyuan Tang, Jianxin Wu, Qing Huang

**Affiliations:** Skin Health and Cosmetic Development & Evaluation Laboratory, China Pharmaceutical University, Nanjing 211198, China

**Keywords:** glabridin, melanogenesis, Wnt/β-catenin, melanocyte dendrite, melanin transfer, Rho family GTPases

## Abstract

**Background**: Glabridin, a natural compound derived from *Glycyrrhiza glabra* L., possesses skin-lightening effects. This study aims to further elucidate the depigmentation mechanism of glabridin by investigating its effects on melanogenesis and melanin transfer. **Methods**: We initially confirmed the anti-melanogenic effects of glabridin in MNT-1 human melanoma cells. Then, we investigated the mechanism of its anti-melanogenic effect by evaluating the protein expression of β-catenin and MITF via Western blot. To investigate melanin transfer, we compared glabridin’s efficacy with that of niacinamide, a recognized inhibitor of melanosome transfer and employed two complementary experimental models: (1) α-melanocyte-stimulating hormone (α-MSH)-stimulated MNT-1 cells to analyze dendrite formation, and (2) a UVB-irradiated co-culture system of MNT-1 cells and HaCaT keratinocytes to evaluate melanin transfer. **Results**: By measuring glabridin’s effects on melanin content, tyrosinase activity, and melanogenesis-related protein expression confirmed its inhibition of melanin synthesis. Further investigation demonstrated that glabridin suppresses melanogenesis by downregulating β-catenin and MITF, indicating inhibition of the Wnt/β-catenin pathway. Furthermore, in α-MSH-treated MNT-1 cells, both glabridin and niacinamide were found to suppress dendrite formation and elongation. In a UVB-exposed co-culture system, both glabridin and niacinamide inhibited melanin transfer to keratinocytes. Mechanistically, these effects were linked to the regulation of Rho GTPases (Rac1, RhoA, Cdc42) and suppression of F-actin reorganization. **Conclusions**: This study provides, for the first time, evidence that the skin-lightening effect of glabridin involves two complementary mechanisms: inhibition of melanogenesis through suppression of the Wnt/β-catenin pathway, and attenuation of both dendricity and melanin transfer via the influence of Rho family GTPases expression.

## 1. Introduction

Melanin is synthesized by basal layer melanocytes, starting from the tyrosine through a series of redox reactions catalyzed by tyrosinase [1]. This process is primarily regulated by three core signaling pathways: cAMP/CREB, Wnt/β-catenin, and MAPK [2]. These pathways converge to activate the microphthalmia-associated transcription factor (MITF) via binding to the M-box in its promoter region. Activated MITF then upregulates the activation of crucial melanogenic enzymes, including tyrosinase (TYR), tyrosinase-related protein 1 (TRP-1), and tyrosinase-related protein 2 (TRP-2), thereby driving melanin synthesis [3]. Among these, the Wnt/β-catenin pathway plays a particularly crucial role in the development of neural crest-derived melanocytes during embryogenesis. In this pathway, the secreted glycoprotein WNT binds to its Frizzled receptors on the cell membrane. This binding leads to the inactivation of glycogen synthase kinase-3β (GSK-3β), resulting in the stabilization and cytoplasmic accumulation of β-catenin. Subsequently, β-catenin translocates into the nucleus, where it forms a transcriptional complex with Lymphoid Enhancer Factor/T-cell Factor (LEF/TCF). This complex potently activates the transcription of the MITF gene [2]. Thus, β-catenin accumulation drives MITF expression, effectively stimulating melanogenesis.

Following its synthesis, the transfer of melanin from melanocytes to surrounding keratinocytes is a critical step in skin pigmentation, which is primarily facilitated by melanocyte dendrites. This process initiates with the maturation and peripheral transport of melanosomes—the specialized organelles responsible for melanin synthesis and storage [1,4]. Upon reaching the cell periphery, melanosomes are delivered into keratinocytes via dendrites of melanocytes [5,6]. Melanocyte dendricity, governed by the coordinated actions of Rho GTPases, is essential for establishing intercellular contact and enabling subsequent melanin transfer [7,8]. Specifically, Ras-related C3 botulinum toxin substrate 1 (Rac1) activation promotes dendrite outgrowth, whereas Ras Homolog Family Member A (RhoA) activation induces their retraction. The balance between these opposing forces fine-tunes dendritic morphology [9,10,11]. Additionally, Cell division cycle protein 42 (Cdc42) contributes to the formation of filopodia—specialized dendritic protrusions that serve as direct contact points with keratinocytes and facilitate melanosome transfer [12,13,14].

Glabridin, a natural isoflavone derived from *Glycyrrhiza glabra* L., exhibits multifaceted bioactivities, including anti-inflammatory, antioxidant, and anti-tumor effects. Notably, glabridin is an established inhibitor of melanogenesis, functioning through the direct inhibition of tyrosinase activity (IC_50_: 0.43 μmol/L) [15] and the suppression of melanin synthesis in B16 melanoma cells via inhibits the cAMP/PKA/CREB/MITF and p38/MAPK signaling pathways [16]. Furthermore, although glabridin has been shown to block Wnt/β-catenin signaling in breast cancer cells [17], its role in melanocyte pigmentation via this pathway has not been investigated.

In this study, we found that glabridin downregulates the expression of β-catenin protein, leading to the subsequent inhibition of MITF expression and ultimately suppressing melanogenesis. Furthermore, glabridin attenuates dendrite formation in melanocytes and prevents melanin transfer to keratinocytes. Mechanistically, these effects are likely mediated through the influence of Rho family GTPases expression and consequent reorganization of the actin cytoskeleton (F-actin). In parallel, we used niacinamide as a positive control. Niacinamide is a recognized inhibitor of melanosome transfer [18,19], although its precise mechanism is not fully elucidated. It exhibited consistent effects in suppressing dendrite elongation and melanin translocation, suggesting it most likely also exerts its effects through the Rho GTPase pathway. Here, we provide for the first time a comprehensive investigation into the role of glabridin in inhibiting melanin synthesis via suppression of the Wnt/β-catenin pathway, and in regulating melanocyte dendrite formation and melanin transfer by influencing the expression of Rho family GTPases. We also discuss the potential mechanism by which niacinamide inhibits melanin transfer.

## 2. Results

### 2.1. Effect of Glabridin on Anti-Melanogenesis

**Figure 1:**
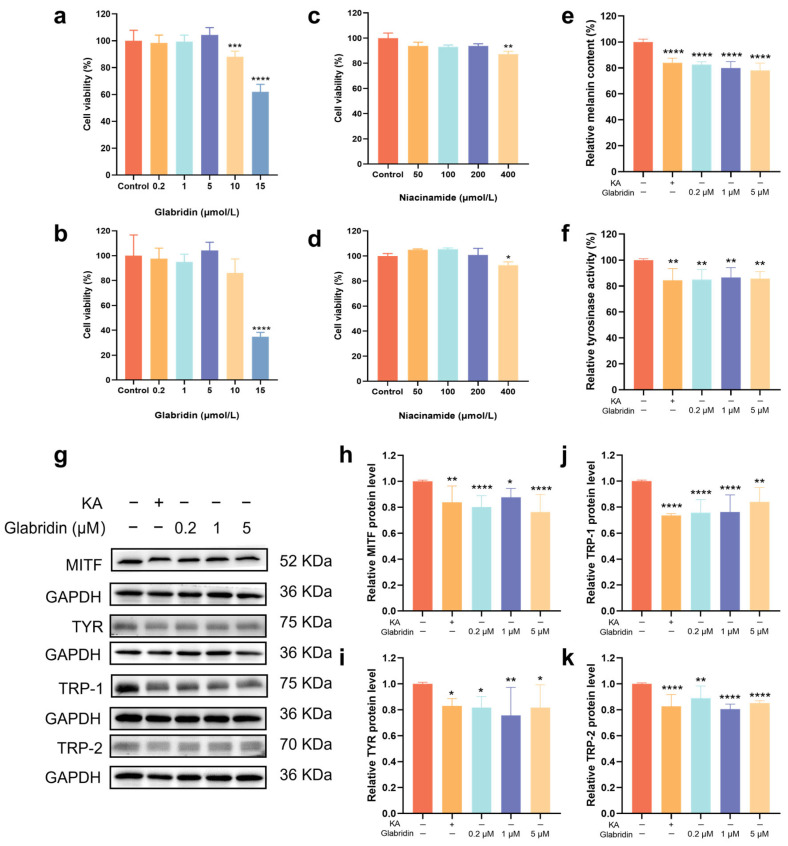
Effect of glabridin on anti-melanogenesis. Different concentrations of glabridin on MNT-1 cell viability (**a**) and on HaCaT cell viability (**b**), assessed by the MTT assay; Different concentrations of niacinamide on MNT-1 cell viability (**c**) and on HaCaT cell viability (**d**), assessed by the MTT assay; (**e**) Glabridin inhibit melanin content in MNT-1 cells. (**f**) Glabridin inhibit intracellular tyrosinase activity in MNT-1 cells. The images of protein bands (**g**) and the relative protein levels of MITF (**h**), TYR (**i**), TRP-1 (**j**) and TRP-2 (**k**). GAPDH served as the internal control. Kojic acid (KA, 200 μg/mL) was used as a positive control. Data are presented as mean ± SD (*n* = 3) (* *p* < 0.05, ** *p* < 0.01, *** *p* < 0.001, **** *p* < 0.0001 vs. control) (one-way ANOVA followed by Tukey’s post hoc test).

To establish safe working concentrations, we evaluated the cytotoxicity of glabridin and niacinamide. Glabridin was non-toxic to MNT-1 cells below 5 μM and to HaCaT cells below 10 μM (Figure 1a,b). Niacinamide demonstrated an excellent safety profile, showing no cytotoxicity at concentrations up to 200 μM (Figure 1c,d). Hence, glabridin at concentrations of 0.2, 1, and 5 μM, and niacinamide at 200 μM were selected for all subsequent experiments.

To investigate the anti-melanogenic effects of glabridin in MNT-1 cells, we evaluated intracellular tyrosinase activity, melanin content, and the protein levels of key melanogenic factors. Glabridin reduced melanin content in a concentration-dependent manner, with reductions of 17.5%, 20.0%, and 21.9% at 0.2, 1, and 5 μM, respectively (Figure 1e) and significantly suppressed intracellular tyrosinase activity (Figure 1f). Consistent with these effects, Western blot analysis confirmed that glabridin markedly downregulated the expression of MITF, TYR, TRP-1, and TRP-2 (Figure 1g–k). The results above demonstrate that glabridin exhibits potent anti-melanogenesis effects by inhibiting the expression of melanogenesis-related proteins and suppressing tyrosinase activity.

### 2.2. Glabridin Inhibits the Wnt/β-Catenin Pathway

**Figure 2:**
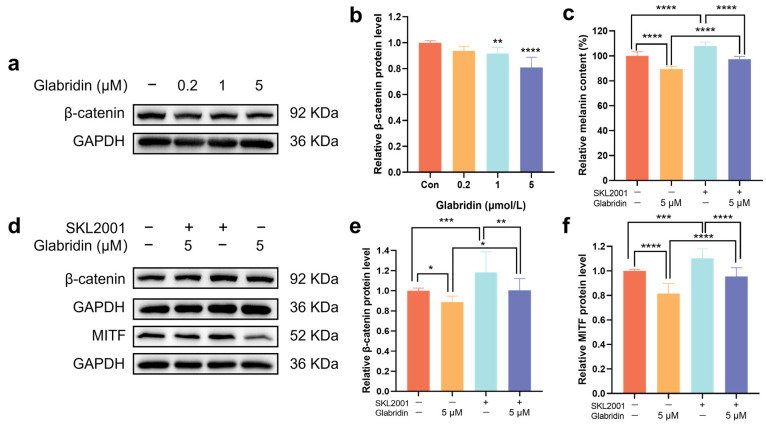
Effects of glabridin on the Wnt/β-catenin pathway. (**a**,**b**) Representative Western blots and quantitative analysis of β-catenin in MNT-1 cells treated with different concentrations of glabridin. (**c**) Melanin content in MNT-1 cells treated with glabridin (with or without SKL2001). (**d**–**f**) Representative Western blots and quantitative analysis of β-catenin and MITF in MNT-1 cells treated with glabridin (with or without SKL2001). GAPDH served as the internal control. Data are presented as mean ± SD (*n* = 3) (**** *p* < 0.0001 vs. control group in (**b**). * *p* < 0.05, ** *p* < 0.01, *** *p* < 0.001, **** *p* < 0.0001 in (**c**,**e**,**f**)) (one-way ANOVA followed by Tukey’s post hoc test).

To investigate the mechanism through which glabridin inhibits melanin synthesis, this study utilized Western blotting to measure the expression levels of β-catenin protein following treatment with varying concentrations of glabridin. The results showed that as the concentration of glabridin increased, the protein expression of β-catenin decreased in a concentration-dependent manner (Figure 2a,b). SKL2001, a specific agonist of the Wnt/β-catenin pathway, enhances intracellular β-catenin levels by inhibiting its phosphorylation at residues Ser33/37/Thr41/Ser45 [20]. First, after stimulating MNT-1 cells with 40 μM SKL2001 alone, the expression levels of β-catenin and its downstream target protein MITF were significantly increased compared to the untreated control group, confirming that SKL2001 effectively activates this pathway. Notably, the inhibitory effects of glabridin on β-catenin and MITF expression was reversed to almost normal levels in the glabridin-treated group when SKL2001 was added simultaneously (Figure 2d–f). Consistent with the Western blot data, the melanin content measurements aligned with the observed protein expression trends (Figure 2c). These collective results indicate that glabridin suppresses melanogenesis by inhibiting the Wnt/β-catenin signaling pathway.

### 2.3. Effect of Glabridin on the Dendrites of MNT-1 Cells

Beyond melanogenesis, skin pigmentation is critically influenced by melanin transfer, a process mediated by melanocyte dendrites [21,22]. To determine whether glabridin affects dendritic morphology, we quantified dendrite length and number in MNT-1 cells. Dendrite length was measured from the nucleus to the tip of individual dendrites in cells with well-defined borders using the Mshot Main software (version 1.1.6); at least 50 cells were measured per condition. The dendritic index, defined as the percentage of cells with three or more dendrites, was assessed from at least 100 cells per group [23].

**Figure 3:**
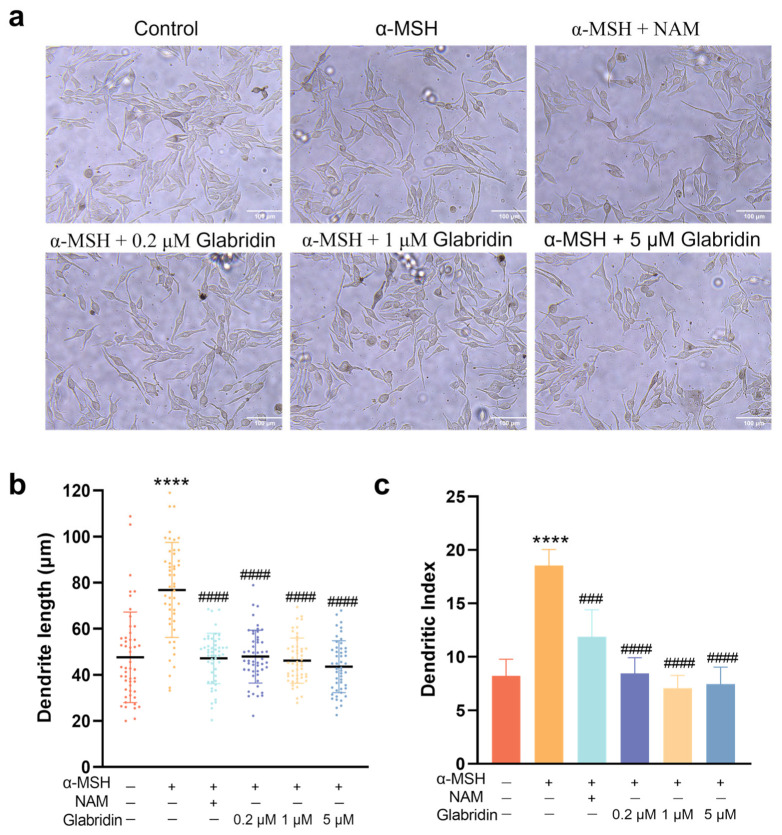
Glabridin inhibits α-MSH-induced dendrite outgrowth in MNT-1 melanocytes. (**a**) Images of MNT-1 cell morphology under brightfield microscopy after treatment with glabridin. Each dot represents an individual cell measurement, and different colors indicate different experimental groups as labeled on the *x*-axis. Niacinamide (NAM, 200 μM) was used as a positive control. Scale bar, 100 μm. (**b**) Quantification of average dendrite length. (**c**) Quantification of the Dendritic Index, defined as the percentage of cells with ≥3 dendrites. Data are represented as mean ± SD (*n* = 3) (**** *p* < 0.0001 vs. control; ### *p* < 0.001 and #### *p* < 0.0001 vs. α-MSH-stimulated group) (one-way ANOVA followed by Tukey’s post hoc test).

Stimulation with α-MSH significantly increased the average dendrite length by 38.8 μm. Glabridin treatment concentration-dependently attenuated this α-MSH-induced elongation, reducing the length by 23.9, 27.4, and 31.6 μm at 0.2, 1, and 5 μM, respectively (Figure 3b). α-MSH also raised the Dendritic Index from 8.2% to 18.5%, and glabridin suppressed this dendritic index to 8.4%, 7.1%, and 7.4% at the corresponding concentrations (Figure 3c). Consistent with these findings, 200 μM niacinamide similarly inhibited both α-MSH-induced dendrite elongation and the increase in dendritic index (Figure 3).

### 2.4. Effect of Glabridin on Melanin Transfer in the MNT-1 and HaCaT Co-Culture System

**Figure 4:**
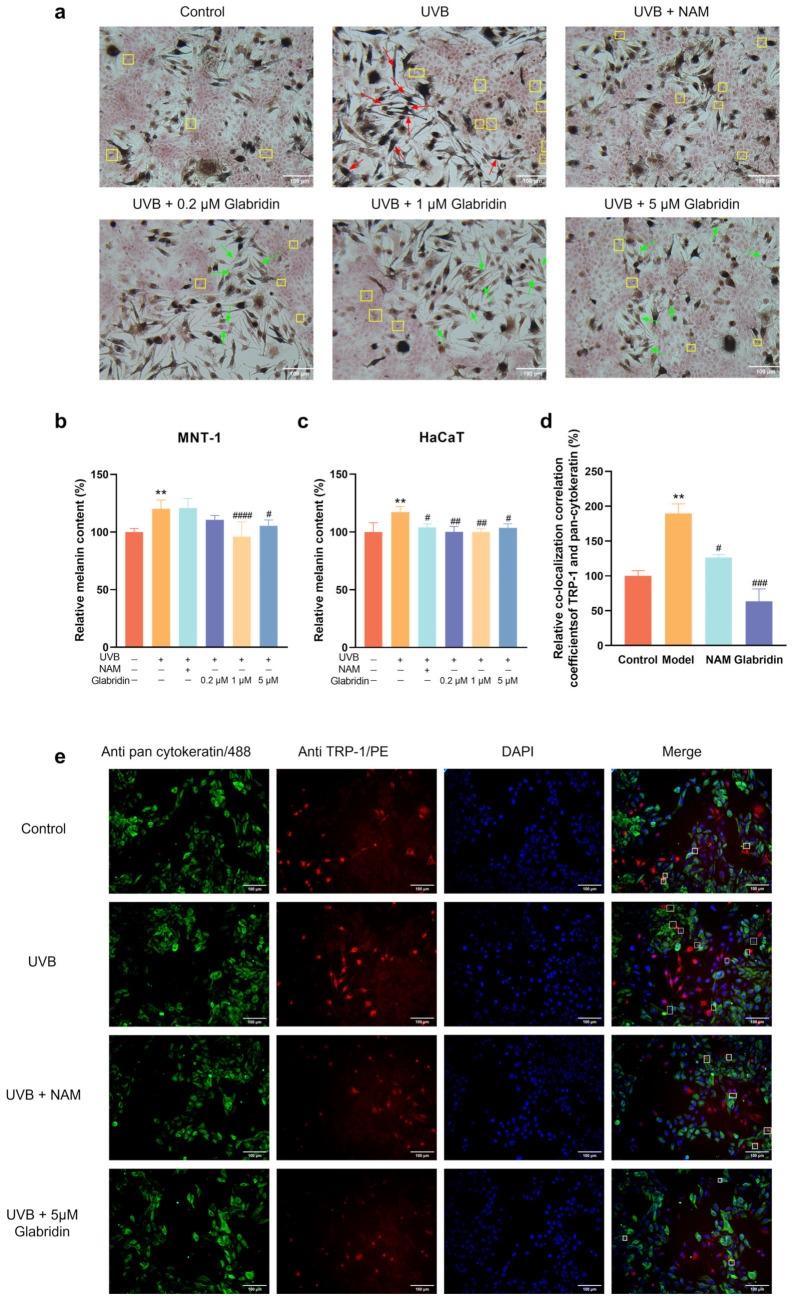
Glabridin inhibits UVB-induced melanin transfer in MNT-1/HaCaT co-culture systems. (**a**) Masson-Fontana staining of the co-culture system. Red arrows: UVB-induced melanocytes with enhanced melanogenesis and elongated dendrites. Green arrows: glabridin-treated melanocytes showing reduced melanin content and shortened dendrites. Yellow boxes: melanosome transferred to HaCaT cells. Niacinamide (NAM, 200 μM) served as a positive control. Scale bar, 100 μm. (**b**,**c**) Melanin content in MNT-1 cells (**b**) and HaCaT cells (**c**) after UVB irradiation followed by treatment with niacinamide and different concentrations of glabridin. (**d**) Quantification of TRP-1/pan-cytokeratin co-localization coefficients. (**e**) Immunofluorescence staining showing TRP-1 (melanosome marker, red) and pan-cytokeratin (keratinocyte marker, green) localization. White boxes indicate regions of co-localization. Scale bar, 100 μm. Data are presented as mean ± SD (*n* = 3) (** *p* < 0.01 vs. the untreated control group; # *p* < 0.05, ## *p* < 0.01, ### *p* < 0.001, and #### *p* < 0.0001 vs. UVB-treated group) (one-way ANOVA followed by Tukey’s post hoc test).

Given that melanin transfer to keratinocytes is a key factor of influencing skin pigmentation, we assessed the impact of glabridin on this process using an MNT-1/HaCaT co-culture model. Masson-Fontana staining revealed that UVB irradiation enhanced melanocyte length and Dendritic Index and promoted melanin transfer to keratinocytes, as indicated by increased perinuclear melanin granules in HaCaT cells (Figure 4a). Glabridin treatment attenuated these changes, lightening melanocyte coloration, shortening dendrites, and reducing melanin granules in keratinocytes.

We further quantified melanin content in both MNT-1 and HaCaT cells under co-culture conditions. After seeding the two cell types at a 1:2 ratio, the co-culture system was irradiated with 5 mJ/cm^2^ UVB. Niacinamide and different concentrations of glabridin were added and cultured for 48 h. MNT-1 and HaCaT cells were separated using 0.25% trypsin, and melanin content was measured separately in each cell type. UVB irritation significantly increased melanin levels in both MNT-1 and HaCaT cells (Figure 4b,c). Glabridin suppressed this increase in both cell types, whereas niacinamide—a known transfer inhibitor [18]—reduced melanin only in HaCaT cells without affecting melanogenesis [19,24].

To directly visualize melanin transfer, we performed immunofluorescence co-staining of the melanosomal marker TRP-1 and keratinocyte marker pan-cytokeratin in the MNT-1/HaCaT co-culture system. Compared to the untreated group, UVB irradiation increased the colocalization correlation coefficient of TRP-1 and pan-cytokeratin by 89.8%, indicating enhanced melanin transfer. This effect decreased by 66.5% upon treatment with 5 μM glabridin and by 33.5% upon treatment with 200 μM niacinamide compared with the UVB irradiation group (Figure 4d,e). Glabridin not only suppresses melanin synthesis but also inhibits melanin transfer by blocking dendrite formation, achieving comprehensive anti-pigmentation effects.

### 2.5. Effects of Glabridin on Rac1, RhoA and Cdc42 mRNA and Protein Expression in MNT-1 Cells

**Figure 5:**
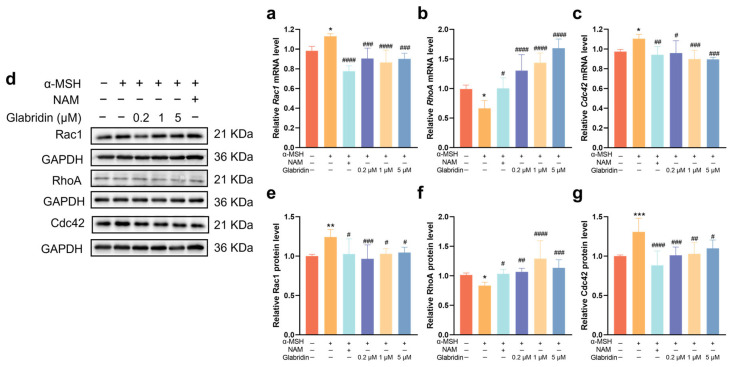
Effect of different concentrations of glabridin on mRNA and protein expression of Rac1, RhoA, and Cdc42 in MNT-1 cells. (**a**–**c**) The mRNA expression of *Rac1*, *RhoA*, and *Cdc42* in MNT-1 cells. (**d**–**g**) Representative Western blots and quantitative analysis of Rac1, RhoA, and Cdc42 in MNT-1 cells. The cDNA for GAPDH served as the internal control. Niacinamide (NAM, 200 μM) was used as a positive control. Data are presented as mean ± SD (*n* = 3) (* *p* < 0.05, ** *p* < 0.01, *** *p* < 0.001 vs. the untreated control group; # *p* < 0.05, ## *p* < 0.01, ### *p* < 0.001, and #### *p* < 0.0001 vs. α-MSH-treated group) (one-way ANOVA followed by Tukey’s post hoc test).

To investigate the mechanism underlying glabridin’s inhibition of dendrite formation, we analyzed mRNA and protein expression of Rho GTPases (Rac1, RhoA, Cdc42), the key regulators of dendritic morphology. Following α-MSH treatment, mRNA expression levels of *Rac1* and *Cdc42* were significantly elevated compared to the control, whereas *RhoA* expression was reduced. Glabridin treatment at 0.2, 1, and 5 μM significantly reversed these α-MSH-induced changes, reducing *Rac1* and *Cdc42* mRNA expression levels and enhancing *RhoA* expression relative to the α-MSH-treated group (Figure 5a–c). Consistent with the mRNA results, protein expression analysis revealed similar trends for all three GTPases (Figure 5d–g). α-MSH stimulation significantly upregulated Rac1 protein expression, which was markedly suppressed by glabridin treatment (Figure 5e). Conversely, α-MSH downregulated RhoA expression, and glabridin concentration-dependently reversed this suppression (Figure 5f). For Cdc42, α-MSH treatment significantly increased Cdc42 protein levels, and glabridin treatment significantly inhibited its expression (Figure 5g).

### 2.6. Effect of Glabridin on F-Actin in MNT-1 Cells

**Figure 6:**
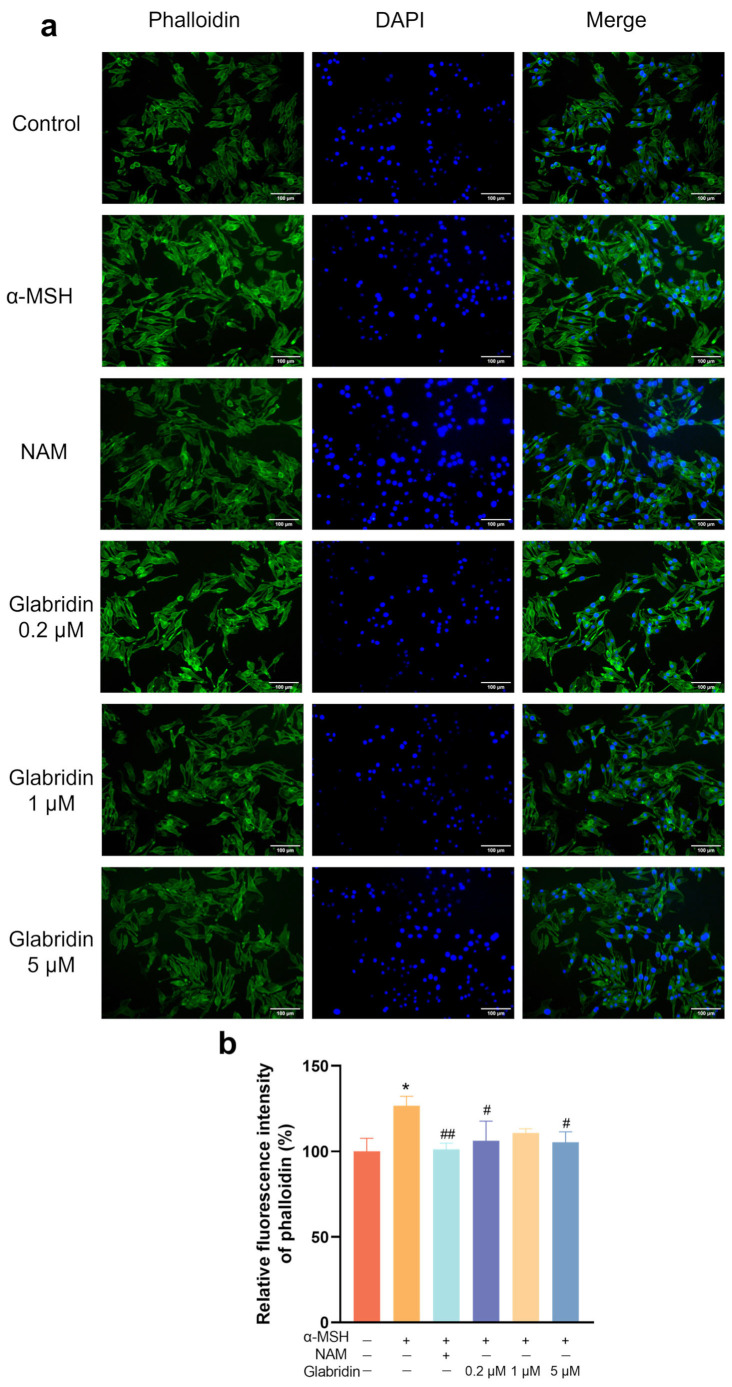
Glabridin suppresses α-MSH-induced F-actin reorganization in MNT-1 cells. (**a**) Representative fluorescence images of phalloidin-stained F-actin. Green fluorescence shows F-actin, while blue fluorescence shows the nuclei of MNT-1 cells. Niacinamide (NAM, 200 μM) was used as a positive control. Scale bar, 100 μm. (**b**) Quantitative analysis of F-actin fluorescence intensity. Data are presented as mean ± SD (*n* = 3) (* *p* < 0.05 vs. the untreated control group; # *p* < 0.05, ## *p* < 0.01 vs. α-MSH-treated group) (one-way ANOVA followed by Tukey’s post hoc test).

The elongation and retraction of dendritic tips are associated with the reorganization of filamentous actin (F-actin) and microtubules [25]. The Rho-GTPases are capable of integrating extracellular signals from diverse receptor types and initiating the rearrangement of F-actin [26,27]. Using rhodamine-phalloidin staining, which specifically labels F-actin, we found that α-MSH stimulated MNT-1 cells significantly increased F-actin fluorescence intensity by 26.68% compared to untreated control group. Glabridin treatment (0.2–5 μM) effectively reversed this increase, reducing fluorescence intensity by 12.52~16.84%. Additionally, niacinamide significantly decreased F-actin fluorescence intensity by 20.05% compared to the α-MSH-treated group (Figure 6). The results indicate that glabridin and niacinamide may inhibit dendrite formation by modulating F-actin cytoskeletal remodeling.

## 3. Discussion

Melanin synthesis within melanocytes and its subsequent transfer to keratinocytes are fundamental processes determining skin pigmentation [28]. Firstly, regarding melanogenesis, it is known that MITF is a downstream target gene of Wnt/β-catenin signaling in melanocytes. Nuclear β-catenin interacts with its co-activates T cell factor (TCF2), activating the TCF/LEF binding to MITF promoter and leading its transcription [2,29]. Interestingly, we observed that glabridin effectively inhibited β-catenin accumulation in MNT-1 cells. Importantly, glabridin almost completely reversed the SKL2001 induced increases in β-catenin and MITF protein expression and melanin content, restoring them to near normal levels. While glabridin has previously been reported to modulate Wnt/β-catenin signaling in breast cancer cells [17], the present study is the first report showing that glabridin may inhibit MITF expression and subsequent melanin synthesis by regulating the Wnt/β-catenin signaling pathway in melanocytes.

The melanin transfer is facilitated by melanocyte dendrites, which are dynamic, hormone-sensitive structures rich in actin and microtubules that deliver melanosomes to surrounding keratinocytes [10]. The content and distribution of melanin within keratinocytes critically influence skin color. The epidermis of black skin has more and larger singly distributed melanosomes in the keratinocytes and corneocytes than that of white skin [30]. Our findings demonstrate that glabridin not only suppresses melanogenesis but also inhibits dendrite formation in melanocytes. Using a UVB-irradiated melanocyte–keratinocyte co-culture system, we further confirmed that glabridin significantly reduces melanin transfer to keratinocytes. To elucidate the mechanism by which glabridin inhibits melanin transfer, we subsequently investigated its effects on Rho family GTPases and actin reorganization. In our study, α-MSH stimulation significantly altered the expression of Rac1, RhoA, and Cdc42, and induced pronounced F-actin reorganization. Compared to the α-MSH-treated group, glabridin treatment significantly suppressed the expression of Rac1 (promotes dendritic growth) and Cdc42 (promotes filopodia formation) at both the gene and protein levels, while increasing the expression of RhoA (stimulates dendritic retraction). Furthermore, compared to the α-MSH-treated group, F-actin reorganization was markedly inhibited. Although Niacinamide is widely used as a melanin transfer inhibitor, its mechanism of action has remained unclear. This study reveals that niacinamide may inhibit melanin transfer by regulating the expression of Rho family GTPases and suppressing F-actin reorganization.

Based on our experimental findings, we propose that glabridin inhibits melanogenesis and melanin transfer through mechanisms involving the Wnt/β-catenin pathway and Rho family GTPases, respectively. Furthermore, we speculate that niacinamide may also exert its inhibitory effects on melanin transfer by modulating the expression of Rho family GTPases; however, this hypothesis requires further investigation and experimental validation by using specific inhibitors or genetic knockdown of Rho GTPases are needed to determine whether they play a causal role in niacinamide’s effects on melanocyte dendricity.

To better contextualize the unique mechanism of glabridin among established skin-lightening agents, we compared its mode of action with that of niacinamide and kojic acid (Table 1).

Unlike kojic acid and niacinamide, which target single steps in pigmentation, glabridin exerts a dual effect by inhibiting both melanogenesis and melanin transfer, offering a multifunctional approach to skin lightening.

Nevertheless, several limitations of this study should be acknowledged, along with directions for future research. First, all experiments were conducted using non-primary cells (MNT-1 and HaCaT cells). Although these cells are well-established and validated models for melanogenesis and skin biology research [32], they may not fully recapitulate the physiological state of normal human skin. Therefore, validation in normal primary human melanocytes, 3D skin models, and in vivo systems is required. Second, while this study primarily examined changes in β-catenin and MITF downstream of the Wnt/β-catenin pathway, as well as expression levels of Rho family GTPases, the upstream molecular targets of glabridin remain unidentified. Future studies employing molecular docking simulations and surface plasmon resonance (SPR) could help identify these direct targets. Third, although we demonstrated that glabridin suppresses the expression of β-catenin and Rho family GTPases, its effects on nuclear β-catenin accumulation and the GTP-bound activation status of Rho GTPases were not directly assessed. Further investigations using nuclear fractionation, luciferase reporter assays, and GST pull-down assays are needed to confirm their functional involvement. Finally, the proposed role of Rho GTPases in glabridin-mediated actin reorganization requires additional validation through genetic manipulation techniques such as siRNA knockdown or CRISPR/Cas9 knockout of RhoA, Rac1, and Cdc42.

Despite these limitations, the present study provides the first evidence that glabridin exerts a dual mechanism in melanocytes, inhibiting melanogenesis via the Wnt/β-catenin pathway and suppressing dendrite formation by influencing the expression of Rho GTPases. This dual action establishes a foundation for future research and potential clinical applications of glabridin as a multifunctional skin-lightening agent.

## 4. Materials and Methods

### 4.1. Cell Culture

Both MNT-1 melanoma (MNT-1) cells and Human keratinocytes (HaCaT) were sourced from Zhejiang Meisen Cell Technology Co., Ltd. (Hangzhou, China). The culture medium for MNT-1 cells consisted of DMEM with 20% FBS, 1% NEAA, 10% AIM-V, and antibiotics, while HaCaT cells were grown in DMEM containing 10% FBS and antibiotics. All cultures were cultured statically in a constant temperature incubator at 37 °C with 5% CO_2_.

### 4.2. Cell Viability Test

Cell viability following glabridin and niacinamide treatment were assessed using the MTT assay. MNT-1 and HaCaT cells were plated in 96-well plates (8 × 10^3^ cells/well) and cultured for 60 h. Then, the cells were exposed to varying concentrations of glabridin (purity ≥ 99%, GuYu Co., Ltd., Guangzhou, China) or niacinamide (purity ≥ 99%, Aladdin, Shanghai, China) for an additional 48 h. Subsequently, the culture supernatant was removed, and the cells were incubated with MTT solution (0.5 mg/mL) at 37 °C for 4 h. The resulting formazan crystals were dissolved in dimethyl sulfoxide (DMSO). Absorbance was measured at 490 nm using a microplate reader. Viability was calculated as a percentage relative to untreated control cells.

### 4.3. Intracellular Tyrosinase Activity Assay

Following a 60 h culture period in 12-well plates at an initial seeding density of 8 × 10^4^ cells/well, MNT-1 cells were treated for 48 h with increasing concentrations of glabridin in 2% FBS-containing medium. To evaluate tyrosinase activity, cells were lysed with RIPA buffer (Solarbio, Beijing, China) and the supernatant was collected for protein quantification using a BCA kit (Beyotime, Shanghai, China). Equal amounts of protein were added to a new 96-well plate, and 100 μL of 10 mM L-DOPA solution was added to each well as the substrate. The reaction mixture was incubated in a 37 °C incubator for 15 min. Absorbance was immediately measured at 475 nm using a microplate reader (Molecular Devices Corporation, San Jose, CA, USA). Results were normalized and expressed as percentage of untreated controls [33].

### 4.4. Measurement of Intracellular Melanin Content

MNT-1 cells were plated at a concentration of 8 × 10^4^ cells/well into 12-well plates, respectively. After 60 h incubation, cells were exposed to various concentrations of glabridin or kojic acid (purity ≥ 99%, Aladdin, Shanghai, China) for 48 h. The experimental concentration of kojic acid was determined based on a previously published article by our research group [34]. At the end of the treatment, after washing with PBS, RIPA lysis buffer was added to each well, and the cells were scraped and collected. The lysate was centrifuged, and the supernatant was discarded, leaving the black pellet. The precipitate was dissolved in 1 M NaOH containing 10% DMSO and heated at 80 °C for 2 h to ensure complete dissolution. Finally, absorbance was read at 405 nm [33].

MNT-1 cells were plated at a density of 8 × 10^4^ cells/well in 12-well plates, respectively. After 60 h incubation, cells were exposed to glabridin and SKL2001 (HY-101085, MCE, Morristown, NJ, USA) for 48 h. Subsequent steps followed the protocol described above.

In the MNT-1 and HaCaT co-culture system, MNT-1 and HaCaT cells were plated at a 1:2 ratio in a 12-well plate and cultured for 36 h. After irradiation with 5 mJ/cm^2^ UVB, niacinamide and different concentrations of glabridin were added and incubated for another 48 h. Subsequently, based on the difference in adhesion capacity between MNT-1 and HaCaT cells (MNT-1 cells exhibit significantly weaker adhesion compared to HaCaT cells) [19], the co-culture was treated with 0.25% trypsin for 3 min. Gentle shaking was applied to suspend the MNT-1 cells, which were then collected. RIPA high-efficiency tissue lysis buffer was applied to HaCaT cells for lysis, and the cells were subsequently collected. The precipitates from both cell types were separately collected and heated in a water bath at 80 °C for 2 h in 1 M NaOH containing 10% DMSO. Finally, absorbance was read at 405 nm. The purity of separated MNT-1 and HaCaT populations was confirmed by morphological assessment under an inverted fluorescence microscope (Supplementary Figure S1).

### 4.5. Masson-Fontana Melanin Staining

MNT-1 and HaCaT cells were seeded at a 1:2 ratio in a 12-well plate and cultured for 36 h. After irradiation with 5 mJ/cm^2^ UVB, niacinamide and different concentrations of glabridin were added and incubated for another 48 h. The co-culture cells were fixed in 4% paraformaldehyde (Biosharp, Beijing, China) for 10 min, then Masson-Fontana melanin staining kit (R20610-3 * 50 mL, Yuanye, Shanghai, China) was used for the experiment. Images were acquired using an inverted fluorescence microscope (Mshot, Guangzhou, China).

### 4.6. Immunofluorescence Staining

MNT-1 and HaCaT cells were seeded at a 1:2 ratio in a 12-well plate and cultured for 36 h. After irradiation with 5 mJ/cm^2^ UVB, 200 μM niacinamide and 5 μM glabridin was added and further cultured for 48 h. The co-cultured cells were rinsed with ice-cold PBS and fixed in 4% paraformaldehyde for 10 min, then washed three times with PBS for 5 min each. Subsequently, the cells were incubated overnight (20 h) at 4 °C with a mixture of anti-TRP1 antibody (sc-166857, Santa Cruz, CA, USA) and anti-pan cytokeratin antibody (2g1e2, Proteintech, Wuhan, China). After removing the primary antibody, the cells were washed three times with PBST (50 mL PBS + 50 μL Tween 20) for 5 min each. A mixture of PE-labeled goat anti-mouse secondary antibody (bs-0296G-PE, Bioss, Beijing, China) and 488-labeled goat anti-rabbit IgG (H + L) (A0243, Beyotime, Shanghai, China) secondary antibody was added in the dark and incubated at room temperature for 1.5 h. After another PBST wash, nuclear staining was performed with DAPI for 5 min. Finally, anti-fade mounting medium was applied to cover the bottom. Observations and image acquisition were performed using an inverted fluorescence microscope. The obtained images were processed and analyzed using Image Pro Plus software (version 6.0.0) [34].

### 4.7. Quantitative Real-Time PCR (RT-qPCR) Analysis

MNT-1 cells were seeded at a density of 8 × 10^4^ cells/well in 12-well plates, respectively. After 60 h incubation, the cells were treated with glabridin and niacinamide in the presence of α-MSH for 36 h. Following two washes with PBS, total RNA was extracted by scraping the cells with RNA-easy isolation reagent. RNA concentration was measured, and RNA was reverse transcribed into cDNA according to the manufacturer’s instructions for the HiScript^®^ III RT SuperMix (Vazyme, Nanjing, China). Finally, target mRNA levels, including RhoA, Rac1, and Cdc42 (Table 2), were quantified using ChamQ SYBR qPCR Master Mix (Vazyme, Nanjing, China) on a BIOER LineGene 9600 Plus real-time PCR system (BIOER, Hangzhou, China).

### 4.8. Western Blot Analysis

MNT-1 cells were seeded at a density of 2 × 10^5^ cells/well in 6-well plates, respectively. After 60 h incubation, In the anti-melanogenesis assay, different concentrations of glabridin and kojic acid were added; as for Wnt/β-catenin pathway, glabridin and SKL2001 (HY-101085, MCE) were supplemented. The cells were then cultured for a further 24 h under these conditions. After incubation, the cells were washed twice with ice-cold PBS, then incubated with ice-cold RIPA lysis buffer for 15 min and scraped to extract proteins. Protein concentrations were determined using a Bicinchoninic Acid (BCA) kit (Beyotime, Shanghai, China), and then diluted with RIPA lysis buffer to the same concentration. Add one-quarter volume of 4 × Loading Buffer, then boil to denature the proteins. Following protein quantification, samples were subjected to electrophoresis on 10% SDS-PAGE gels, and the resolved proteins were transferred to PVDF membranes by electroblotting. Subsequently, the membranes were incubated overnight (12 h) at 4 °C with antibodies against β-catenin (51067-2-AP, Proteintech, Wuhan, China), MITF (ET1702-86, HuaBio, Hangzhou, China) and GAPDH (60004-1-lg, Proteintech, Wuhan, China).

While in the Rho GTPase protein assay, the wells were treated with glabridin and niacinamide in the presence of 1 μg/mL α-MSH, followed by a further incubation of 48 h. Subsequent steps followed the protocol described above. Briefly, membranes were blocked using QuickBlock™ Blocking Buffer for 15 min, after which they were incubated overnight (12 h) at 4 °C with antibodies against MITF (ET1702-86, HuaBio, Hangzhou, China), TYR (sc-20035, Santa Cruz, CA, USA), TRP-1 (sc-166857, Santa Cruz, CA, USA), TRP-2 (sc-74439, Santa Cruz, CA, USA), Rac1 (sc-514583, Santa Cruz, CA, USA), RhoA (sc-418, Santa Cruz, CA, USA), Cdc42 (ET1701-7, HuaBio, Hangzhou, China) and GAPDH (60004-1-lg, Proteintech, Wuhan, China). Subsequently, the membranes were incubated with horseradish peroxidase-conjugated secondary antibodies for 1 h at room temperature. After incubation, the membranes were washed three more times with TBST for 10 min each. Finally, ECL substrate was added, and protein bands were detected using a chemiluminescence imaging system (Tanon, Shanghai, China). Band intensities were analyzed using Image J software (version 1.8.0) [35].

### 4.9. Phalloidin Staining

After incubating MNT-1 cells with α-MSH and glabridin and niacinamide for 48 h, the cells were stained with phalloidin (F-actin, C8082-50T, Adamas Life, Shanghai, China) following the manufacturer’s instructions. Images were captured using an inverted fluorescence microscope (Mshot, Guangzhou, China) and processed using Image J software. Subsequently, we used the Image J software to measure the fluorescence intensity of phalloidin in the images and performed normalization with the blank control group as the baseline to obtain the relative fluorescence intensity.

### 4.10. Statistical Analysis

One-way ANOVA followed by Tukey’s post hoc test was used to compare differences between groups, and statistical significance was set at *p* < 0.05. All experiments were performed in triplicate (as independent biological replicates), and data are presented as mean ± SD. The GraphPad Prism software (version 8.0.2) was used for statistical analysis and graph plotting.

## 5. Conclusions

In conclusion, this study shows that glabridin exerts its anti-melanogenesis effects by modulating the Wnt/β-catenin pathway and inhibits melanocyte dendrite formation through regulating the expression levels of Rho family GTPases, ultimately suppressing melanin transfer to keratinocytes. Overall, these results demonstrate glabridin as a multi-targeted depigmenting agent that acts across three critical stages of skin pigmentation: melanin synthesis, dendritic formation, and intercellular transfer. This study expands the mechanistic understanding of glabridin and supports its potential application in treating hyperpigmentation disorders.

## Figures and Tables

**Table 1:** Whitening ingredients and their mechanisms of action.

Agent	Mechanism
Glabridin	Inhibit melanogenesis via cAMP/PKA/CREB/MITF and p38/MAPK pathways [16], Wnt/β-catenin/MITF pathway. Inhibit melanin transfer via Rho family GTPase-mediated dendritic formation suppression.
Niacinamide	Inhibit melanin transfer [18]
Kojic acid	Inhibit melanogenesis by suppressing tyrosinase activity [31]

**Table 2:** RT-qPCR prime.

Gene	Sequence
GAPDH	Forward 5′-GCACCGTACCGGCTGAGAAC-3′
	Reverse 5′-ATGGTGGTGAAGACGCCAGT-3′
Rac1	Forward 5′-AAGAGAAAATGCCTGCTGTTGTAA-3′
	Reverse 5′-GCGTACAAAGGTTCCAAGGG-3′
RhoA	Forward 5′-CATCCGGAAGAAACTGGT-3′
	Reverse 5′-TCCCACAAAGCCAACT-3′
Cdc42	Forward 5′-GCTGTCAAGTATGTGGAGTGTT-3′
	Reverse 5′-GCGGCTCTTCTTCGGTTCT-3′

## Data Availability

The original contributions presented in this study are included in the article/Supplementary Material. Further inquiries can be directed to the corresponding authors.

## Supplementary Materials

The following supporting information can be downloaded at: https://www.mdpi.com/article/10.3390/ph19030469/s1, Figure S1: Morphological validation of MNT-1 and HaCaT cell separation by differential adhesion.
