# Supplementary material for: Glabridin Inhibits Melanogenesis and Melanin Transfer via Wnt/β-Catenin Pathway and Rho Family GTPase-Mediated Dendritic Formation Suppression
Source: Pharmaceuticals (Basel). 2026 Mar 12;19(3):469. doi: 10.3390/ph19030469 (PMC13028802; doi:10.3390/ph19030469)
Supplement: Supplementary file 1 [file pharmaceuticals-19-00469-s001.zip › pharmaceuticals-4161705-supplementary.pdf]

Supplementary Information(S1)

Glabridin Inhibits Melanogenesis and Melanin Transfer via Wnt/β-catenin Pathway and Rho Family GTPase-Mediated Dendritic Formation Suppression

The purity of separated MNT-1 and HaCaT populations was confirmed by morphological assessment under an inverted fluorescence microscope. As shown in the supplementary figure, no MNT-1 cells are present in the post-separation images, and HaCaT cells maintain their normal morphology, demonstrating that this method reliably separates MNT-1 cells from the co-culture without affecting HaCaT morphology.

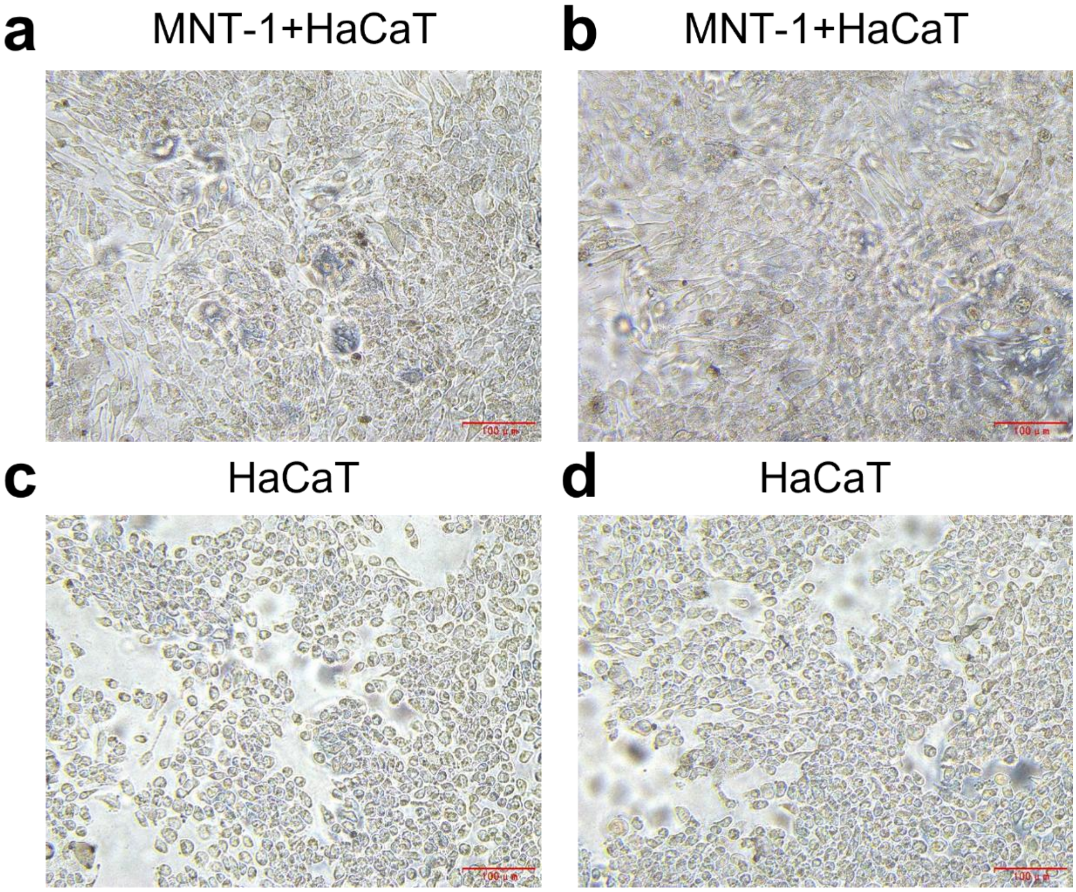

Supplementary Figure S1. Morphological validation of MNT-1 and HaCaT cell separation by differential adhesion. (a, b) The co-culture before separation, in which spindle-shaped, fibroblast-like cells are MNT-1 cells, while cobblestone-shaped, adherent cells in clusters are HaCaT cells. (c, d) HaCaT cells remaining after detachment of MNT-1 cells from the co-culture shown in panels a and b, retaining their cobblestone morphology. Scale bar = 100 μm.
